# Repair of spinal cord injury by implantation of bFGF-incorporated HEMA-MOETACL hydrogel in rats

**DOI:** 10.1038/srep09017

**Published:** 2015-03-12

**Authors:** Bo Chen, Jianyu He, Hao Yang, Qian Zhang, Lingling Zhang, Xian Zhang, En Xie, Cuicui Liu, Rui Zhang, Yi Wang, Linhong Huang, Dingjun Hao

**Affiliations:** 1 Translational Medicine Center, Hong Hui Hospital, Xi'an Jiaotong University College of Medicine, Xi'an, 710054 China; 2 Department of Pharmacology, Xi'an Jiaotong University College of Medicine, Xi'an, 710061 China; 3 School of Advanced Materials and Nanotechnology, Xidian University, Xi'an, 710126 China; 4 Department of Spine Surgery, Hong Hui Hospital, Xi'an Jiaotong University College of Medicine, Xi'an, 710054 China

**Keywords:** Spinal cord injury, Drug delivery

## Abstract

There is no effective strategy for the treatment of spinal cord injury (SCI). An appropriate combination of hydrogel materials and neurotrophic factor therapy is currently thought to be a promising approach. In this study, we performed experiments to evaluate the synergic effect of implanting hydroxyl ethyl methacrylate [2-(methacryloyloxy)ethyl] trimethylammonium chloride (HEMA-MOETACL) hydrogel incorporated with basic fibroblast growth factor (bFGF) into the site of surgically induced SCI. Prior to implantation, the combined hydrogel was surrounded by an acellular vascular matrix. Sprague–Dawley rats underwent complete spinal cord transection at the T-9 level, followed by implantation of bFGF/HEMA-MOETACL 5 days after transection surgery. Our results showed that the bFGF/HEMA-MOETACL transplant provided a scaffold for the ingrowth of regenerating tissue eight weeks after implantation. Furthermore, this newly designed implant promoted both nerve tissue regeneration and functional recovery following SCI. These results indicate that HEMA-MOETACL hydrogel is a promising scaffold for intrathecal, localized and sustained delivery of bFGF to the injured spinal cord and provide evidence for the possibility that this approach may have clinical applications in the treatment of SCI.

## Introduction

Traumatic spinal cord injury (SCI) is a devastating condition characterized by extensive tissue degeneration and severe neurological dysfunction. There is no effective treatment for this condition. Clinical treatment may involve intravenous administration of the steroid methylprednisolone; however, the efficacy of this approach has been widely disputed^1^. Several clinical trials have also been completed using pharmacological agents^2,3^; however, no trials have shown effective regulation of axonal regeneration. Tissue engineering techniques, which are focused on promoting the regeneration of injured nervous tissue, continue to assume greater importance in neuroscience.

The use of various biomaterials, which is one of the most common tissue engineering techniques, has been shown to be effective in the treatment of SCI^4,5,6,7^. Hydrogels are nontoxic, chemically inert synthetic 3-dimensional polymer biomaterials with pore sizes ranging from 10 to 100 μm and high water content (70–90%). Previous findings have shown that positively charged HEMA-based hydrogel is characterized by extensive ingrowth of connective tissue elements compared with negatively charged HEMA-based hydrogel^8^. The HEMA-MOETACL hydrogel has been proved to carry a positive electric charge. The diffusion properties of this hydrogel indicate that the macroporous structure is available for cell ingrowth and that the diffusion of small cations is minimally hindered within the hydrogel^9^. In addition, a study on its subcutaneous and intracerebral biocompatibility has demonstrated that HEMA-MOETACL hydrogel is biologically inert^10^. Thus, this material is a suitable candidate for biomedical application in SCI treatment.

Blood flow in the spinal cord is also acutely impacted after SCI when vasoconstriction occurs due to factors that are released after immediate cell death as well as through the process of angiogenesis. bFGF is a member of the fibroblast growth factors (FGFs) which regulate a variety of biological functions including neuroprotection, vasodilation, stimulating angiogenesis and suppressing cell apoptosis^11,12,13^. bFGF is abundantly expressed in the nervous system, where it has vital roles and was previously shown to support the survival and growth of neurons and neural stem cells in vitro^14^. After SCI, seriously injured blood vessels leak blood and fluid into the parenchyma, leading to a large pseudocyst cavity and bFGF can decrease immediate vasoconstriction of blood vessels in the tissue near the epicenter of the injury and promote angiogenesis^15^. Previous findings have also shown that bFGF infusion or transgene therapy may promote the regeneration of neurons and axons^16,17^. Thus, we hypothesize that bFGF has the potential to limit degeneration resulting from ischemia and promote angiogenesis following SCI.

In this study, the goal of tissue engineering was to identify the most appropriate combination of factors that would contribute to providing the best scaffold. Therefore we designed a novel bFGF/HEMA-MOETACL transplant which could deliver bFGF to the injured spinal cord in a localized and sustained manner. The HEMA-MOETACL hydrogel incorporated bFGF by polyion complexation and prior to implantation, this combined hydrogel was surrounded by an acellular vascular matrix which consisted mainly of glycosaminoglycan (GAG), elastic and collagen fibers^18,19,20,21^. The unimmunogenic GAG can absorb growth factors and cytokines, thus promoting the adhesion and migration of cells; elastic and collagen fibers can provide mechanical support for the ingrowth of tissue inside the hydrogel. This bFGF/HEMA-MOETACL transplant was implanted into the lesion 5 days after transection surgery. Our results indicate that the bFGF/HEMA-MOETACL transplant is a promising scaffold for nerve tissue regeneration and functional recovery of essentially paralyzed hindlimbs in SCI.

## Results

### Structural characteristics of HEMA-MOETACL hydrogel and acellular vascular matrix

**Figure 1:**
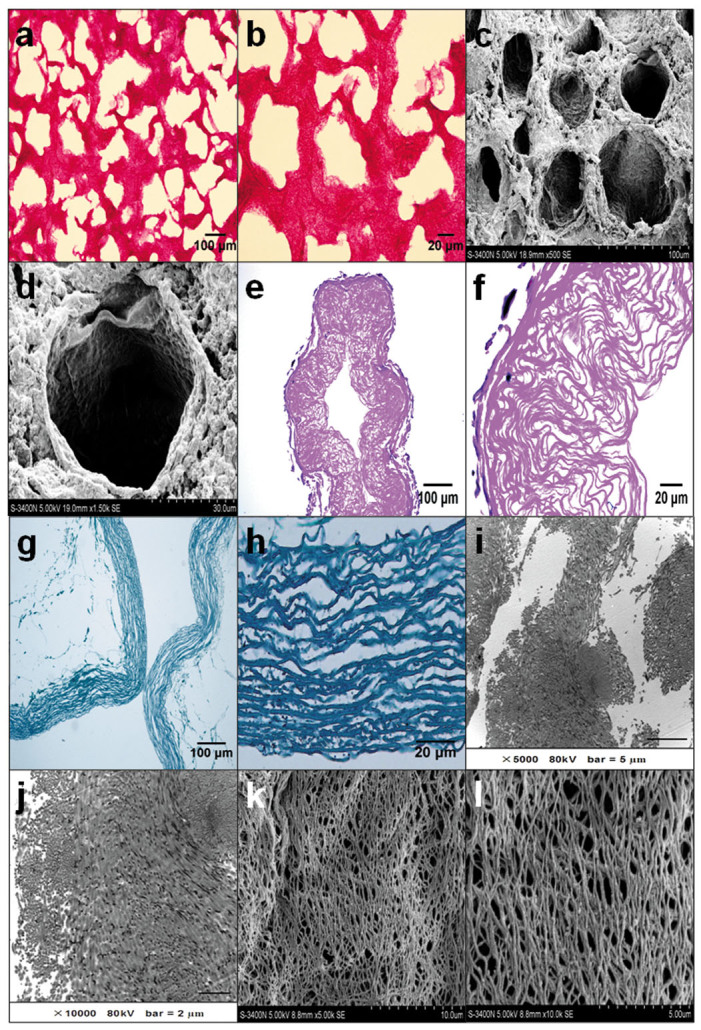
Structure of the HEMA-MOETACL hydrogel and acellular vascular matrix. (a) Semi-thin cross-sections of hydrogel stained with HE. A light microphotograph shows the macroporous structure of the hydrogel. (b) Higher magnification of the macroporous structure. (c) A scanning electron microphotograph shows the ultrastructure of the hydrogel. (d) Higher magnification of the macroporous structure. (e) Semi-thin cross-sections of the acellular vascular matrix stained with HE. A light microphotograph shows the reticulate structure of the acellular vascular matrix. (f) Higher magnification of the reticulate structure. (g) Semi-thin cross-sections of the acellular vascular matrix with Pollak trichrome connective tissue stain. A light microphotograph shows that the elastic fibers and collagen fibers were well conserved. (h) Higher magnification of the elastic and collagen fibers. (i) TEM observation showed no breakage of elastic and collagen fibers after decellularization. (j) Higher magnification of the elastic and collagen fibers. (k) SEM observation demonstrated the ultrastructure of the well conserved fibers. (l) Higher magnification of the reticulate structure.

Semi-thin cross-sections of hydrogel were stained with HE. Representative images showed that the macroporous structure of hydrogel was formed by washing out the particles of the solid porogen, NaCl (Figure 1a, b). The ultrastructure of hydrogel was investigated using SEM. Representative micrographs showed that the macroporous hydrogel had communicating pores (2 × 10^6^ pores per cm^3^) with an average size of 80 μm and a specific pore volume of 0.45 (Figure 1c, d).

Semi-thin cross-sections of acellular vascular matrix were stained with HE. Representative images showed that the cell components were completely removed from rabbit thoracic aorta. The remaining elastic and collagen fibers were arranged in a reticular formation (Figure 1e, f). The histological appearance of elastic and collagen fibers in acellular vascular matrix was assessed by Pollak trichrome connective tissue stain. Representative micrographs showed that the cells were completely removed and collagen fibers were stained blue. The elastic fibers and collagen fibers were well conserved (Figure 1g, h). TEM observations indicated no breakage of elastic and collagen fibers or basement membrane after decellularization (Figure 1i, j). SEM micrographs of acellular vascular matrix demonstrated that the fibers were well conserved with a fine reticulate and porous arrangement after decellularization (Figure 1k, l).

### Functional recovery after bFGF/HEMA-MOETACL implantation

**Figure 2:**
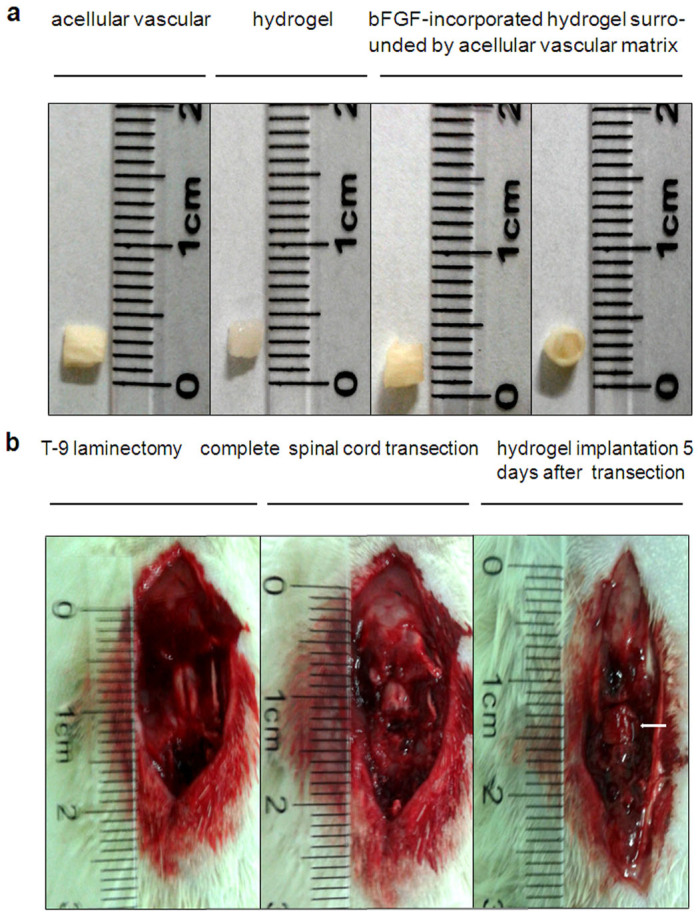
The SCI procedure in rats transplanted with bFGF/HEMA-MOETACL. (a) Preparation of the bFGF/HEMA-MOETACL transplant. The hydrogel was cut into blocks approximately 2 × 2 × 2 mm and the acellular vascular matrix was cut into 2-mm segments. After incorporation of bFGF, the hydrogel block was surrounded by a segment of the acellular vascular matrix. (b) Five days after transection surgery, the bFGF/HEMA-MOETACL transplant (indicated by white arrows) was implanted into the lesion.

To evaluate the effect of bFGF/HEMA-MOETACL implantation on SCI, we used the animal model described above (Figure 2a, b). The behavior of SCI rats in the lesion-control group (n = 8), bFGF-alone group (n = 10), HEMA-MOETACL-alone group (n = 10) and HEMA-MOETACL + bFGF group (n = 12) was tested once a week for eight weeks after implantation. All SCI rats underwent two assessments to evaluate the effect on behavioral recovery.

**Figure 3:**
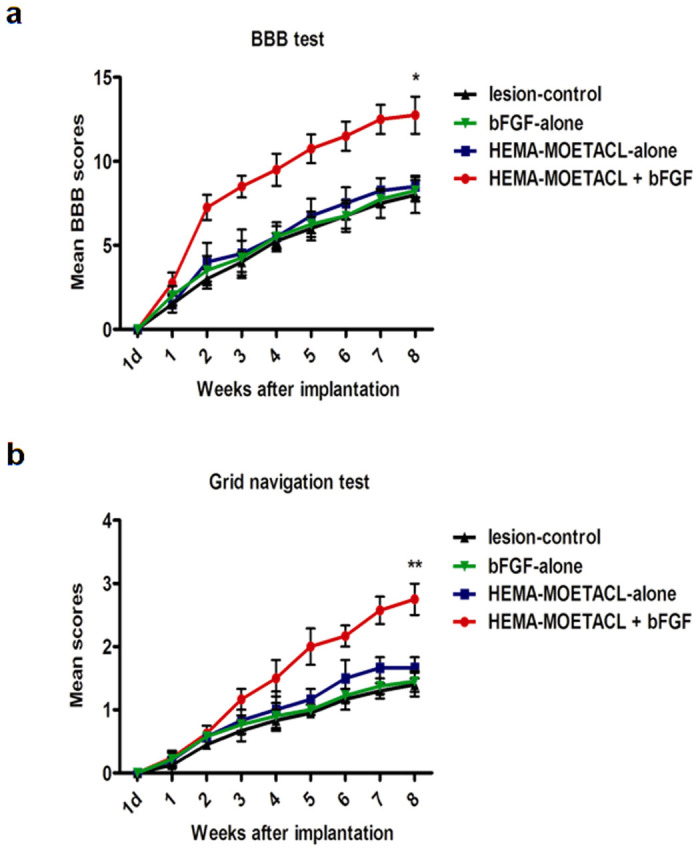
Behavioral improvement after bFGF/HEMA-MOETACL implantation. (a) Locomotor skills were monitored by the BBB rating scale. Eight weeks after implantation, the mean BBB score in the HEMA-MOETACL + bFGF group was significantly higher than that in the other three groups (mean ± SEM, *p < 0.05 versus the HEMA-MOETACL-alone group, *p < 0.05 versus the bFGF-alone group, *p < 0.05 versus the lesion-control group, Student's *t-*test). (b) Sensorimotor skills were monitored by the grid navigation test. Eight weeks after implantation, the mean score in the HEMA-MOETACL + bFGF group was significantly higher than that in the other three groups (mean ± SEM, *p < 0.05 versus the HEMA-MOETACL-alone group, **p < 0.01 versus the bFGF-alone group, **p < 0.01 versus the lesion-control group, Student's *t-*test).

Locomotor skills were assessed by the open field test using the BBB rating scale (Figure 3a). The mean BBB scores over time were higher in the HEMA-MOETACL + bFGF group than in the other three groups. Eight weeks after implantation, the mean BBB score in the HEMA-MOETACL + bFGF group was 12.7 ± 0.6, which was significantly higher than that in the other three groups (*p* < 0.05 versus the HEMA-MOETACL-alone group, *p* < 0.05 versus the bFGF-alone group, *p* < 0.05 versus the lesion-control group). The HEMA-MOETACL + bFGF group showed a significant improvement in hindlimb performance eight weeks after implantation. Moreover, only this group reached and overtook the weight-supporting step level (score of 9). Interestingly, locomotor scores in the HEMA-MOETACL + bFGF group were distinguishable from the other groups one week after implantation. The lesion-control group scored 0 on day 1 after transection surgery and the score gradually increased to a final mean score of 8.0 ± 0.5 eight weeks after surgery. Eight weeks after implantation, the mean BBB score was not significantly increased in both the HEMA-MOETACL-alone group (8.5 ± 0.3) and the bFGF-alone group (8.2 ± 0.3) compared with the lesion-control group (*p* > 0.05). There was no obvious improvement in hindlimb performance in both the HEMA-MOETACL-alone group and the bFGF-alone group. Sensorimotor skills were monitored by the grid navigation test (Figure 3b). Even when SCI rats with bFGF/HEMA-MOETACL implantation did not recover their toe control and the capacity to realize fine movements, their mean scores over time were significantly improved. Eight weeks after implantation, the mean score in the HEMA-MOETACL + bFGF group was 2.8 ± 0.1, which was significantly higher than that in the other three groups (*p* < 0.05 versus the HEMA-MOETACL-alone group, *p* < 0.01 versus the bFGF-alone group, *p* < 0.01 versus the lesion-control group). The lesion-control group scored 0 on day 1 after transection surgery and the score gradually increased to a final mean score of 1.4 ± 0.1 eight weeks after surgery. Eight weeks after implantation, the mean score was not significantly increased in both the HEMA-MOETACL-alone group (1.7 ± 0.1) and the bFGF-alone group (1.5 ± 0.1) compared with the lesion-control group (*p* > 0.05).

Neural conduction in all SCI rats was tested by SSEPs and MEPs eight weeks after implantation. Electrophysiological measurements of SSEPs and MEPs, which were analyzed by onset latencies and peak amplitudes, were used to estimate whether axons conducting sensory and/or motor information crossed the injury site.

**Figure 4:**
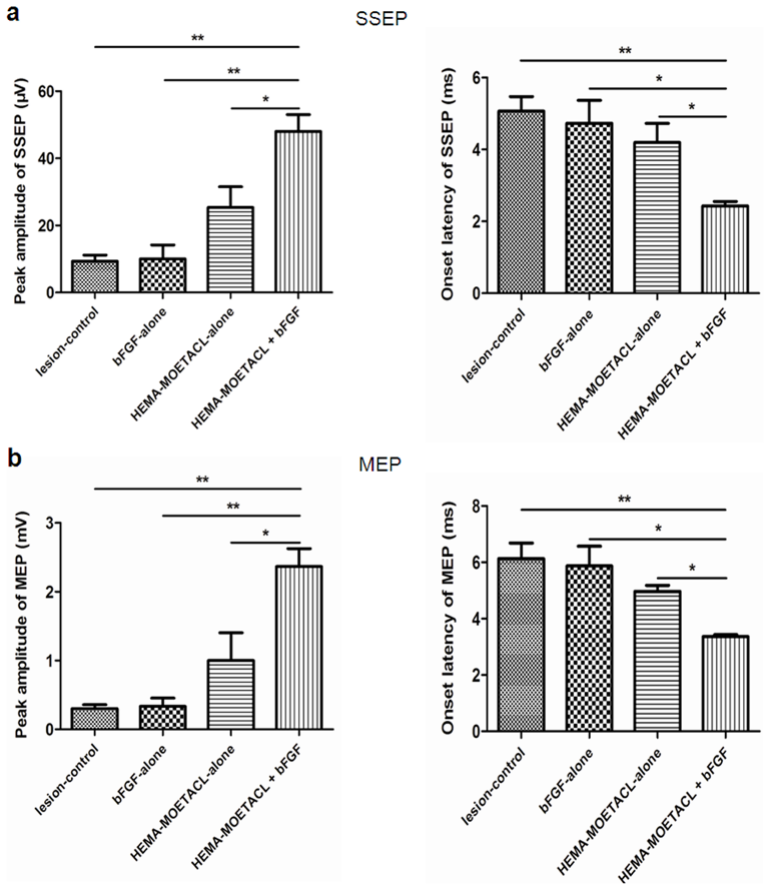
Electrophysiological improvement after bFGF/HEMA-MOETACL implantation. Eight weeks after implantation, neural conduction in all SCI rats was assessed by SSEPs and MEPs, which were analyzed by onset latencies and peak amplitudes. (a) Peak amplitudes and onset latencies of SSEPs. The peak amplitudes of SSEPs were significantly higher in the HEMA-MOETACL + bFGF group than in the other three groups (mean ± SEM, *p < 0.05 versus the HEMA-MOETACL-alone group, **p < 0.01 versus the bFGF-alone group, **p < 0.01 versus the lesion-control group, Student's *t*-test). The onset latencies of SSEPs were significantly shorter in the HEMA-MOETACL + bFGF group than in the other three groups (mean ± SEM, *p < 0.05 versus the HEMA-MOETACL-alone group, *p < 0.05 versus the bFGF-alone group, **p < 0.01 versus the lesion-control group, Student's *t*-test). (b) Peak amplitudes and onset latencies of MEPs. The peak amplitudes of MEPs in the HEMA-MOETACL + bFGF group were significantly higher than those in the other three groups (mean ± SEM, *p < 0.05 versus the HEMA-MOETACL-alone group, **p < 0.01 versus the bFGF-alone group, **p < 0.01 versus the lesion-control group, Student's *t*-test). The onset latencies of MEPs in the HEMA-MOETACL + bFGF group were significantly shorter than those in the other three groups (mean ± SEM, *p < 0.05 versus the HEMA-MOETACL-alone group, *p < 0.05 versus the bFGF-alone group, **p < 0.01 versus the lesion-control group, Student's *t*-test).

The SSEPs were recorded in the sensorimotor cortex following stimulation of the sciatic nerve (Figure 4a). The peak amplitudes of SSEPs were significantly higher in the HEMA-MOETACL + bFGF group than in the other three groups (*p* < 0.05 versus the HEMA-MOETACL-alone group, *p* < 0.01 versus the bFGF-alone group, *p* < 0.01 versus the lesion-control group). In both the HEMA-MOETACL-alone group and the bFGF-alone group, the peak amplitudes of SSEPs were not significantly higher compared with those in the lesion-control group (*p* > 0.05). The onset latencies of SSEPs were significantly shorter in the HEMA-MOETACL + bFGF group than in the other three groups (*p* < 0.05 versus the HEMA-MOETACL-alone group, *p* < 0.05 versus the bFGF-alone group, *p* < 0.01 versus the lesion-control group). In both the HEMA-MOETACL-alone group and the bFGF-alone group, the onset latencies of SSEPs were not significantly shorter compared with those in the lesion-control group (*p* > 0.05). The MEPs were recorded in the sciatic nerve after stimulation of the motor cortex (Figure 4b). After SCI, animals may show lengthened MEP onset latencies and reduced peak amplitudes. The peak amplitudes of MEPs in the HEMA-MOETACL + bFGF group were significantly increased than those in the other three groups (*p* < 0.05 versus the HEMA-MOETACL-alone group, *p* < 0.01 versus the bFGF-alone group, *p* < 0.01 versus the lesion-control group). In both the HEMA-MOETACL-alone group and the bFGF-alone group, the peak amplitudes of MEPs were not significantly increased compared with those in the lesion-control group (*p* > 0.05). The onset latencies of MEPs in the HEMA-MOETACL + bFGF group were significantly shorter than those in the other three groups (*p* < 0.05 versus the HEMA-MOETACL-alone group, *p* < 0.05 versus the bFGF-alone group, *p* < 0.01 versus the lesion-control group). In both the HEMA-MOETACL-alone group and the bFGF-alone group, the onset latencies of MEPs were not significantly shorter compared with those in the lesion-control group (*p* > 0.05). Therefore, the improvements in behavior and electrophysiology demonstrated that the injured axons may recover to direct the action potentials following bFGF/HEMA-MOETACL implantation.

### Bridging the spinal cord lesion with bFGF/HEMA-MOETACL implantation

**Figure 5:**
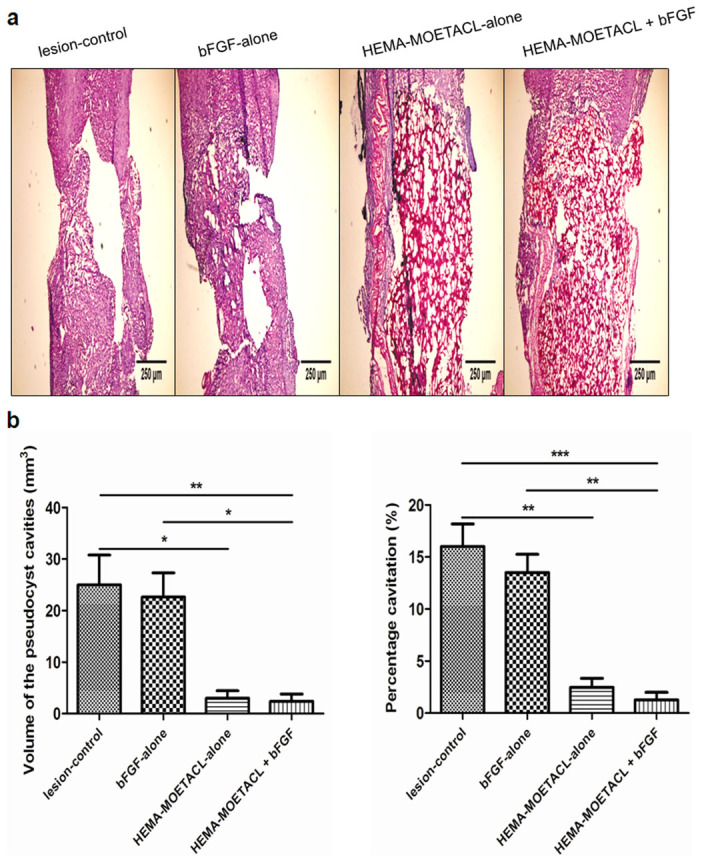
Reduced cavitation after bFGF/HEMA-MOETACL implantation. For measurements of the cavity volume and the percentage cavitation, HE sections were examined as detailed in Materials and methods. (a) Representative images of longitudinal sections of injured spinal cords eight weeks after implantation. (b) Quantitative analysis of the cavity volume and percentage cavitation on the basis of histological longitudinal images. The volume of the pseudocyst cavities was significantly reduced in the HEMA-MOETACL + bFGF group than in the lesion-control group and the bFGF-alone group (mean ± SEM, **p < 0.01 and *p < 0.05, respectively, Student's *t*-test). The volume of the pseudocyst cavities was significantly reduced in the HEMA-MOETACL-alone group than in the lesion-control group (mean ± SEM, *p < 0.05, Student's *t*-test). Analogously, the percentage cavitation was significantly decreased in the HEMA-MOETACL + bFGF group than in the lesion-control group and the bFGF-alone group (mean ± SEM, ***p < 0.001 and **p < 0.01, respectively, Student's *t*-test). The percentage cavitation was significantly decreased in the HEMA-MOETACL-alone group than in the lesion-control group (mean ± SEM, **p < 0.01, Student's *t*-test).

All SCI rats were killed eight weeks after implantation and longitudinal sections obtained were examined. In both the lesion-control group and the bFGF-alone group, the lesion site consisted primarily of a major pseudocyst, which had developed gradually to form a mechanical obstacle between the stumps of the spinal cord (Figure 5a). In both the HEMA-MOETACL + bFGF group and the HEMA-MOETACL-alone group, the hydrogel formed a bridge between both stumps of the spinal cord and the integrity of the spinal cord was restored, as shown in the Figure 5. The mechanical properties of the hydrogel allowed it to adhere to the spinal cord stumps without the need for excessive pressure on the tissue. Even when the hydrogel was firmly adhered to the spinal cord tissue, few or no pseudocyst cavities were found at the implant–tissue borders. The volume of the pseudocyst cavities was significantly reduced in the HEMA-MOETACL + bFGF group (2.41 ± 0.80 mm^3^) than in the lesion-control group (25.00 ± 3.33 mm^3^) and the bFGF-alone group (22.67 ± 2.69 mm^3^) (*p* < 0.01 and *p* < 0.05, respectively). The volume of the pseudocyst cavities was significantly reduced in the HEMA-MOETACL-alone group (3.00 ± 0.84 mm^3^) than in the lesion-control group (*p* < 0.05). Analogously, the percentage cavitation was significantly decreased in the HEMA-MOETACL + bFGF group (1.28 ± 0.40%) than in the lesion-control group (16.00 ± 1.26%) and the bFGF-alone group (13.50 ± 1.01%) (*p* < 0.001 and *p* < 0.01, respectively). The percentage cavitation was significantly decreased in the HEMA-MOETACL-alone group (2.48 ± 0.51%) than in the lesion-control group (*p* < 0.01). Moreover, the cavity volume and percentage cavitation in the HEMA-MOETACL + bFGF group tended to decrease compared with those in the HEMA-MOETACL-alone group, however, no significant between-group difference was observed (*p* > 0.05). In the bFGF-alone group, the cavity volume and percentage cavitation were not significantly decreased compared with those in the lesion-control group (*p* > 0.05). A summary of these data and the quantitative analysis are shown in Figure 5b. These results demonstrated a significant reduction in cavitation after implantation of bFGF/HEMA-MOETACL in SCI.

**Figure 6:**
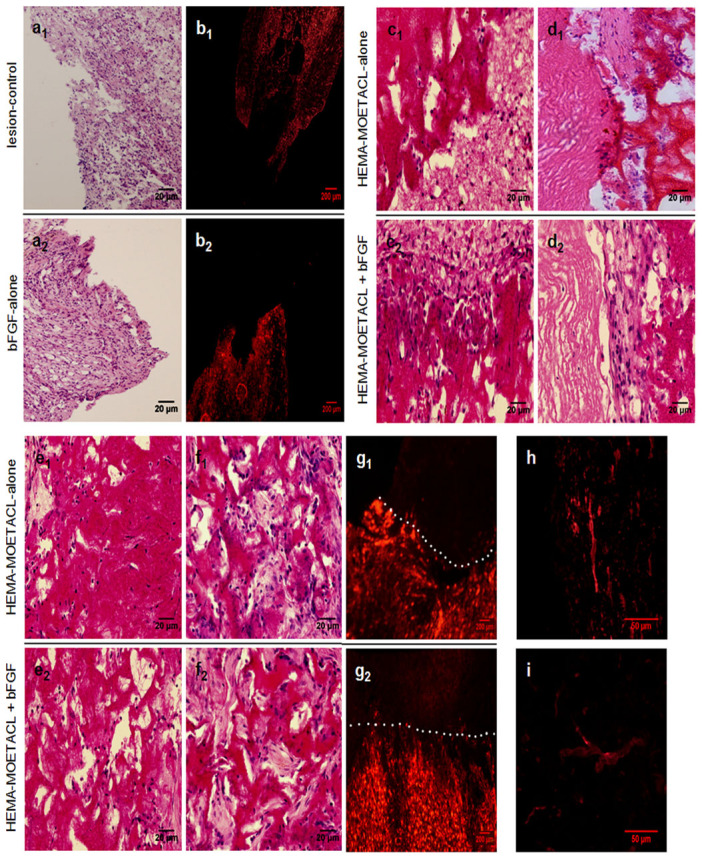
Bridging the SCI following bFGF/HEMA-MOETACL implantation. For histological evaluation of hydrogel integration, longitudinal sections were examined with both light and fluorescence microscopy as detailed in Materials and methods. (a_1_, a_2_) In both the lesion-control group and the bFGF-alone group, the pseudocyst cavity dominated the lesion with a sharp border between the pseudocyst cavity and the residual tissue. (b_1_, b_2_) GFAP-positive astrocytes formed a glial scar around the pseudocyst cavity. (c_1_, c_2_) In both the HEMA-MOETACL + bFGF group and the HEMA-MOETACL-alone group, the hydrogel adhered well to the spinal cord. (d_1_, d_2_) The hydrogel was surrounded by the acellular vascular matrix, which was conserved with a reticulate and porous arrangement. (e_1_, e_2_) No foreign body reactions were found in the hydrogel. (f_1_, f_2_) Connective tissue elements infiltrated the pores of the hydrogel. (g_1_, g_2_) GFAP-positive astrocytes barely crossed the hydrogel–spinal cord boundary. No astrocytes grew inside the hydrogel. The white dotted lines indicate the boundary between the hydrogel and spinal cord tissue. (h) NF 160-positive neurofilaments were detected in the hydrogels from the HEMA-MOETACL + bFGF group and the HEMA-MOETACL-alone group. (i) In both the HEMA-MOETACL + bFGF group and the HEMA-MOETACL-alone group, RECA-1-positive blood vessel endothelial cells were also detected in the hydrogels.

In both the lesion-control group and the bFGF-alone group, the pseudocyst cavity dominated the lesion without hydrogel implantation, with a sharp border between the pseudocyst cavity and the residual tissue (Figure 6a_1_, a_2_). In addition, GFAP-positive astrocytes formed a glial scar around the pseudocyst cavity (Figure 6b_1_, b_2_). The integrity of the spinal cord was severely damaged by the pseudocyst and scar formation. In both the HEMA-MOETACL + bFGF group and the HEMA-MOETACL-alone group, the hydrogel adhered well to the spinal cord (Figure 6c_1_, c_2_). Both groups showed good hydrogel integration inside the lesion site although few or no pseudocyst cavities were found at the implant–tissue borders. The hydrogel was surrounded by the acellular vascular matrix, which improved mechanical support for the ingrowth of tissue inside the hydrogel and promoted cell adhesion and growth. The remaining elastic and collagen fibers were conserved with a reticulate and porous arrangement, which allowed infiltration of cerebrospinal fluid (Figure 6d_1_, d_2_). Cellular infiltration in the hydrogel pores was observed. We specifically looked for any inflammatory response or adverse tissue reaction to the scaffold and no foreign body reactions were found in the hydrogel (Figure 6e_1_, e_2_). Connective tissue elements infiltrated the pores of the hydrogel (Figure 6f_1_, f_2_). GFAP-positive astrocytes barely crossed the hydrogel–spinal cord boundary. The hydrogel mechanically blocked the invasion of scar tissue into the lesion site (Figure 6g_1_, g_2_). A few astrocytic processes were found on the boundary. NF 160-positive neurofilaments (Figure 6h) and RECA-1-positive blood vessel endothelial cells (Figure 6i) were detected inside the hydrogel in both the HEMA-MOETACL + bFGF group and the HEMA-MOETACL-alone group. Axons infiltrated the hydrogel pores from both the proximal and distal ends of the lesion and grew inside the hydrogel. Blood vessels grew and branched inside the hydrogel. Moreover, general tissue morphology was similar in the rats in all groups, where no proliferative lesions were observed due to bFGF delivery (data not shown). On the hydrogel–spinal cord boundary, astrocytes formed a thin layer of a dense network, constituting a glial scar. This was not obviously different from glial scarring in the lesion-control group, suggesting that astrocytic processes were not influenced by bFGF delivery from the bFGF/HEMA-MOETACL transplant.

### Stimulative regeneration of axons and blood vessels after bFGF/HEMA-MOETACL implantation

**Figure 7:**
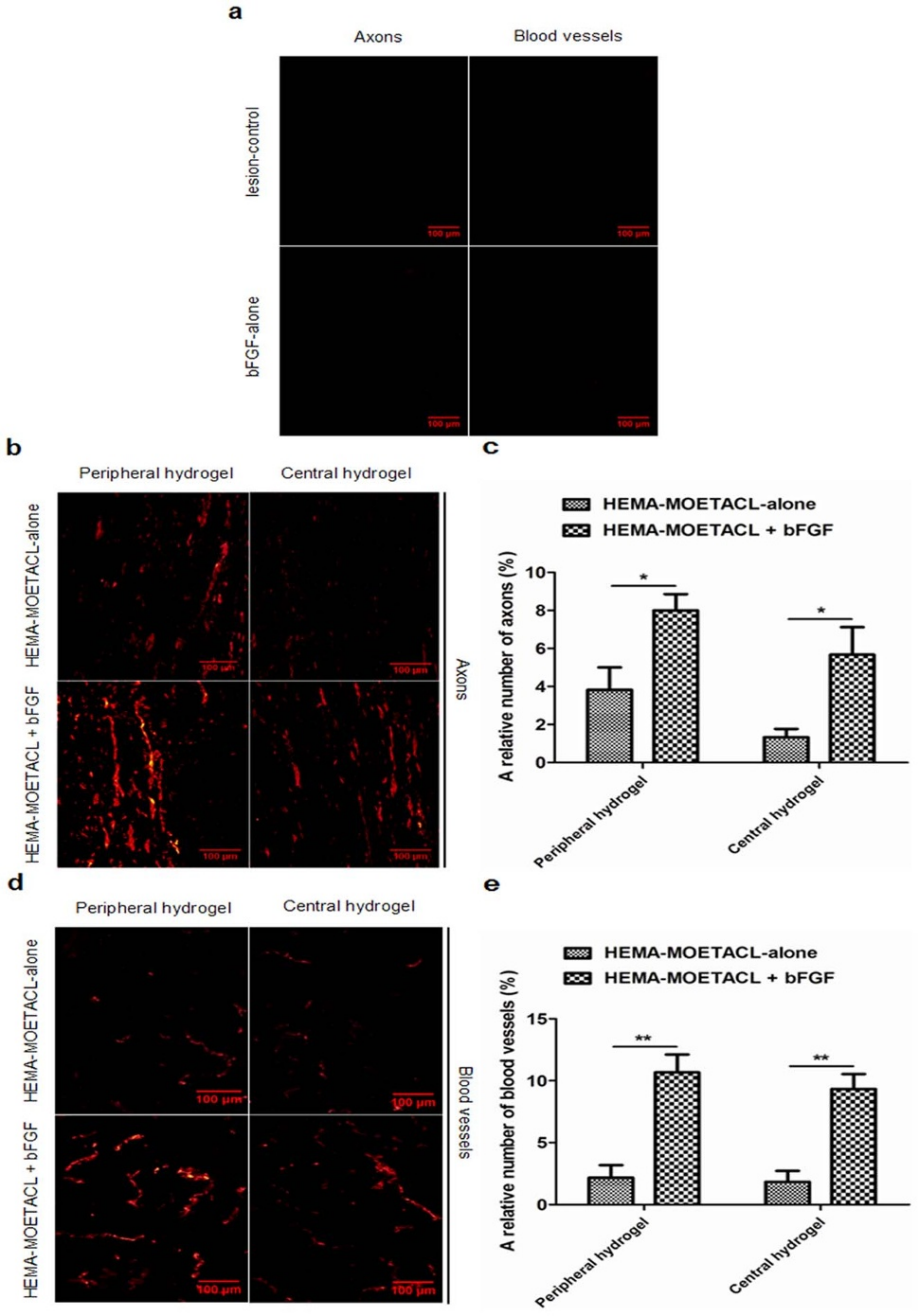
Increased ingrowth of axons and blood vessels in the peripheral and central zones of the hydrogel after bFGF/HEMA-MOETACL implantation. To calculate the ingrowth of axons and blood vessels eight weeks after implantation, NF 160-positive neurofilaments and RECA-1-positive blood vessel endothelial cells inside the hydrogel were examined in longitudinal sections using a fluorescence microscope as detailed in Materials and methods. (a) In both the lesion-control group and the bFGF-alone group, NF 160-positive neurofilaments and RECA-1-positive blood vessel endothelial cells were hardly detected in the lesion site. (b) Representative images show neurofilament staining in the peripheral and central zones of the hydrogel. (c) Quantitative analysis of the relative number of axons. Both in the peripheral and central zones of the hydrogel, the relative number of axons was significantly higher in the HEMA-MOETACL + bFGF group than in the HEMA-MOETACL-alone group (mean ± SEM, *p < 0.05 and *p < 0.05, respectively, Student's *t*-test). (d) Representative images show staining of blood vessel endothelial cells in the peripheral and central zones of the hydrogel. (e) Quantitative analysis of the relative number of blood vessels. Analogously, both in the peripheral and central zones of the hydrogel, the relative number of blood vessels was significantly higher in the HEMA-MOETACL + bFGF group than in the HEMA-MOETACL-alone group (mean ± SEM, **p < 0.01 and **p < 0.01, respectively, Student's *t*-test).

To calculate the ingrowth of axons and blood vessels inside the hydrogel eight weeks after implantation, we examined the positive staining of neurofilaments and blood vessel endothelial cells and divided this by the area of the hydrogel in longitudinal sections. The lesion-control group and the bFGF-alone group showed severe tissue defects, pseudocyst cavities, without any cellular infiltration (Figure 7a). In both the HEMA-MOETACL + bFGF group and the HEMA-MOETACL-alone group, axons infiltrated the peripheral and central zones of the hydrogel (Figure 7b). In the peripheral zones of the hydrogel in the HEMA-MOETACL + bFGF group, the relative number of axons was 8.00 ± 0.50%, which was significantly higher than that in the HEMA-MOETACL-alone group with 3.83 ± 0.67% (*p* < 0.05). In the central zones of the hydrogel, the relative number of axons in the HEMA-MOETACL + bFGF group and the HEMA-MOETACL-alone group tended to decrease (5.67 ± 0.84%, 1.33 ± 0.25%, respectively). There were significant differences between the two groups (*p* < 0.05). Furthermore, the difference between the two groups increased in the central zones of the hydrogel. A summary of these data and the quantitative analysis are shown in Figure 7c. As shown in Figure 7d, the ingrowth of blood vessels was similar to the ingrowth of axons in the peripheral and central zones of the hydrogel. In the peripheral zones of the hydrogel in the HEMA-MOETACL + bFGF group, the relative number of blood vessels was 10.67 ± 0.84%, which was significantly higher than that in the HEMA-MOETACL-alone group with 2.17 ± 0.59% (*p* < 0.01). An analogous situation to the periphery was observed in the central zones of the hydrogel, where again the relative number of blood vessels was significantly higher in the HEMA-MOETACL + bFGF group (9.33 ± 0.69%) compared with the HEMA-MOETACL-alone group (1.83 ± 0.51%) (*p* < 0.01). The difference between the two groups tended to increase in the central zones of the hydrogel. A summary of these data and the quantitative analysis are shown in Figure 7e. Therefore, implantation of bFGF/HEMA-MOETACL may stimulate the regeneration of axons and blood vessels in SCI.

## Discussion

Bridging a spinal cord lesion with a scaffold is one of the most crucial steps in SCI treatment. Methacrylate-based hydrogels have been proved to be partially effective in SCI repair by bridging the tissue defect^22,23,24,25^. The physical and chemical properties of these hydrogels can be tailored to construct a biomaterial for tissue regeneration. Several physical properties have been evaluated in some studies, such as stiffness^26,27^, the size of the pores^28^ and the surface charge^22^. In this study, we prepared HEMA-MOETACL hydrogel which has been shown to carry a positive electric charge. Furthermore, concerning the site of injury, experimental lesions are most commonly performed at the thoracic level. However, this is not true for clinical SCI, where cervical lesions are the most common type of injury^29^. In the thoracic region, the key is to restore the long white matter tracts, which would force the need for hydrogel with longitudinally oriented pores. While, in the cervical region, both sprouting from the neighboring neural elements and the effect of local circuits have a significant impact on functional outcome. Therefore, the structure of the hydrogel should take into account the longitudinal cranio-caudal direction of long tracts as well as the more perpendicular direction of local circuits. In the present study, we chose HEMA-MOETACL hydrogel when considering the “clinical feasibility” of hydrogel in SCI treatment and the labyrinth spider web-like macroporous structure of this hydrogel was formed.

The HEMA-MOETACL hydrogel can bridge posttraumatic spinal cord pseudocyst cavities^23^. The implantation of this bridging hydrogel alone may not have an effect on behavioral improvement in association with scaffold implantation such as has been found in association with stem cells or neurotrophic factors implantation^23,30,31,32^. Furthermore, a comparative study of four methacrylate hydrogels indicated that the HEMA-MOETACl hydrogel was the best carrier of mesenchymal stem cells (MSCs) in vitro and in vivo^29^. The HAMC hydrogel and the composite of HAMC/PLGA have also been shown to be excellent delivery systems where biomolecules are delivered directly to the injured spinal cord^15,33^. These findings indicate that a suitable hydrogel, combined with appropriate neurotrophic factors or stem cells seems to be a promising approach in the treatment of SCI. In our study, in both the HEMA-MOETACL + bFGF group and the HEMA-MOETACL-alone group, the hydrogel bridged the spinal cord lesion and cellular infiltration in the hydrogel pores was observed. Both groups showed significant reductions in cavitations. However, the HEMA-MOETACL-alone group did not show obvious functional recovery compared to the lesion-control group. We further demonstrated that the bFGF/HEMA-MOETACL transplant may significantly improve functional recovery compared to the HEMA-MOETACL-alone transplant. These results were supported by the histological findings which demonstrated that the implantation of bFGF/HEMA-MOETACL may significantly increase the ingrowth of axons and blood vessels compared to the implantation of HEMA-MOETACL-alone following SCI. Our findings indicate that the bFGF/HEMA-MOETACL transplant may be preferable to the HEMA-MOETACL hydrogel.

Previous studies have indicated the trend in therapeutic strategies using bFGF in the treatment of SCI^11,32,34^. Systemic delivery is the most common drug administration route in clinical practice, however, this approach is not suitable for bFGF as it does not cross the blood–spinal cord barrier and its mitogenic potential may lead to malignant tumors^35^. In addition, systemic bFGF delivery causes vasodilation by opening ATP-sensitive potassium channels and enhancing the release of nitric oxide^36^, while, systemic vasodilation can reduce blood pressure, which is not ideal after SCI. Localized drug delivery, such as implantable catheters and minipump devices, have been used to avoid these issues. However, implantable catheters and minipumps may cause scarring and compression of the spinal cord^37^. To overcome these shortcomings, in the present study, biocompatible HEMA-MOETACL hydrogel was used as a vehicle for the delivery of bFGF. We demonstrated that the transplant was soft and filled the transection cavity with excellent adherence, which may not exert pressure on the surrounding spinal cord tissue. At the hydrogel–spinal cord boundary, astrocytes constituted a glial scar, which was not obviously different from scar tissue in the lesion-control group, suggesting that bFGF delivery by this transplant may not affect scarring. We also looked for adverse tissue reactions to the scaffold. A foreign body reaction, which was previously found following hydrogel implantation^22^, was not observed after bFGF/HEMA-MOETACL implantation. Besides, dose-response experiments have not been performed using bFGF to investigate the dosage necessary to produce angiogenesis in spinal cord tissue, thus we delivered a lower dose of bFGF than that in previous minipump/catheter delivery studies to prevent excess proliferative lesions^38^. These results suggest that the bFGF/HEMA-MOETACL transplant is suitable for the delivery of bFGF to the injured site.

Although bFGF has been reported for its important role in SCI, there was no obvious improvement in the bFGF-alone group compared to the lesion-control group in this study. This was probably due to the limitation that the injection of bFGF-alone 5 days after transection surgery may not achieve sustained delivery of bFGF to the injured spinal cord. This drug delivery approach limited the function of bFGF in SCI treatment. However, the HEMA-MOETACL hydrogel incorporated bFGF by polyion complexation, thus delivering bFGF to the injured spinal cord in a slow and sustained manner. We further demonstrated that the implantation of bFGF/HEMA-MOETACL 5 days after transection may significantly improve both nerve tissue regeneration and functional recovery compared to the injection of bFGF-alone 5 days after transection. These results suggest that this transplant is effective for localized and sustained delivery of bFGF to the injured site.

The timing of implantation is also crucial. A previous study in rats showed that delaying hydrogel implantation until 1 week after SCI may be more effective than immediate hydrogel implantation after injury as a further decrease in the volume of pseudocyst cavities was observed^23^. Also in clinical practice, the implantation of a scaffold during acute posttraumatic surgical stabilization may not be beneficial^39,40^. Taking the advantages of delayed implantation into consideration, in this study, the implantation of hydrogel into the lesion was delayed after transection surgery. In addition, the optimal timing of bFGF delivery in SCI treatment remains unclear, although data on this topic have been provided. Previous studies showed a significant up-regulation of bFGF mRNA and protein expression 3 days after SCI, especially bFGF mRNA, which initially increased 3 days post-injury, then declined and returned to baseline levels at 21 days^41,42^. These results indicate that up-regulation of bFGF gradually declines 3 days post-injury, from which we deduce that in terms of timing, it is advantageous to deliver bFGF to the lesion after up-regulation of bFGF has peaked. In addition, a previous study showed that endogenous adaptive angiogenic processes occur within the first 5–7 days post-injury^43^ and thus therapies intended to improve angiogenesis after SCI should target this time frame. On the basis of these results, we inserted a bFGF/HEMA-MOETACL transplant 5 days after SCI and demonstrated that the implantation of bFGF/HEMA-MOETACL 5 days after transection surgery may significantly promote the ingrowth of blood vessels inside the hydrogel compared to the implantation of HEMA-MOETACL-alone 5 days after transection surgery. A higher density of vasculature usually results in significant functional recovery in SCI^44^, which is similar to the behavioral and electrophysiological improvements after bFGF/HEMA-MOETACL implantation in our study. These results suggest that the improvements following bFGF/HEMA-MOETACL implantation are due to the localized and sustained delivery of bFGF to the injured spinal cord 5 days after transection surgery, which encompasses the time frame of post-traumatic angiogenesis to stimulate the angiogenic response and local blood flow at the lesion site. Previous studies have also demonstrated that implantation of bone marrow stem cells may promote axonal regeneration and functional recovery after SCI^30,31^. To further improve regeneration quality and facilitate the total recovery of lost function, a combination of methods, such as the use of hydrogel seeded with bFGF-transfected bone marrow stem cells, should be considered in SCI treatment.

In summary, the goal of this study was to identify the most appropriate combination of hydrogel and neurotrophic factor which would provide the best scaffold in SCI treatment. We demonstrated that the bFGF/HEMA-MOETACL transplant is a promising scaffold for localized and sustained delivery of bFGF to the injured spinal cord. Eight weeks after bFGF/HEMA-MOETACL implantation, increased ingrowth of regenerating axons and blood vessels was observed in the hydrogel, leading to functional recovery of the essentially paralyzed hindlimbs. These beneficial effects, together with the biological safety and other properties of the materials used to construct the hydrogel, suggest that this approach may have clinical applications in the treatment of SCI.

## Methods

### Synthesis of the hydrogel

Macroporous hydrogel based on HEMA with MOETACl was prepared by radical copolymerization of monomers (HEMA 0.67 g, MOETACl 0.12 g and ethylene dimethacrylate 0.019 g as cross-linker) in the presence of fractionated particles of NaCl with a diameter of 50–90 μm (10.02 g) and the solvent polyethylene glycol (MW 400, 3.79 g), using the initiator 2,2′-azo-bis-isobutyronitrile (0.0067 g) for 8 h at 80°C^45^. After polymerization the hydrogel was washed with water and physiological saline solution once a day for 5 days in order to remove unreacted monomers and solvent. The hydrogel thus obtained was autoclaved (120°C, 20 min) and cut into blocks approximately 2 × 2 × 2 mm (under sterile conditions).

### Preparation of the acellular vascular matrix

An eight-week-old male New Zealand white (NZW) rabbit was sacrificed by injection of air into the ear vein, the thoracic aorta was then harvested under sterile conditions and subsequently immersed in phosphate-buffered saline (PBS) solution using aseptic techniques in a laminar flow hood. The adventitia was stripped off with forceps and the remaining tissue was placed into a 10 mL centrifuge tube and 8 mL of 1.25 g/L trypsin containing 100 U/mL penicillin and 100 U/mL streptomycin was added. The mixture was incubated for 4 h, washed 3 times with PBS and incubated in aseptic deionized water for 12 h. After that, the remaining thoracic aorta media was incubated in 1% (v/v) Triton X-100 containing 100 U/mL penicillin and 100 U/mL streptomycin for 24 h, then washed 3 times with PBS and incubated in aseptic deionized water for 4 h. Finally, the remaining media was incubated in 10 g/L sodium dodecyl sulfate (SDS) containing 100 U/mL penicillin and 100 U/mL streptomycin for 24 h, followed by washing 3 times with PBS and incubating in aseptic deionized water for 4 h. Note that all the above incubation processes were performed in a water bath shaker at 37°C^21,46^. The acellular vascular matrix was sterilized by exposure to 10 kGy ^60^Co-γ radiation after vacuum freeze-drying processes and cut into 2-mm segments (under sterile conditions).

### Morphology analysis

HEMA-MOETACL hydrogel was visualized under scanning electron microscopy (SEM; S-3400N, HITACHI, Tokyo, Japan). Semi-thin (thickness: 10 μm) cross-sections of the hydrogel were stained with hematoxylin-eosin (HE) and examined under a light microscope. Rabbit thoracic aorta-derived acellular vascular matrix was visualized under SEM (S-3400N, HITACHI, Tokyo, Japan). Semi-thin (thickness: 10 μm) and ultra-thin (thickness: 50.0 nm) sections of the acellular vascular matrix were prepared. Semi-thin cross-sections were stained with HE and Pollak's trichrome and examined under a light microscope. Ultra-thin sections were stained with uranyl acetate and lead citrate and examined by transmission electron microscopy (TEM; H-600, HITACHI, Tokyo, Japan). Morphology evaluations were conducted by two independent examiners who were blind to the experimental design.

### Preparation of bFGF/HEMA-MOETACL hydrogel

Under sterile conditions, recombinant human bFGF (2 μg; Sigma–Aldrich, St. Louis, MO, USA) was dissolved in 100 μL of aseptic artificial cerebrospinal fluid (aCSF; 148 mM NaCl, 3 mM KCl, 1.4 mM CaCl_2_, 0.8 mM MgCl_2_, 1.5 mM Na_2_HPO_4_, 0.2 mM NaH_2_PO_4_; pH 7.4) containing 20 mg/mL of heparin (Sigma Chemical Co., St. Louis, MO, USA) and the obtained drug solution was incubated overnight at 4°C^15,47,48^. The dried hydrogel block was impregnated with the drug solution and incubated at 37°C for 1 h to prepare the HEMA-MOETACL hydrogel incorporating bFGF by polyion complexation. Prior to implantation, this combined hydrogel block was surrounded by a segment of the acellular vascular matrix. The final dose of bFGF contained in this hydrogel transplant was 2 μg.

### Hydrogel implantation and animal care

A complete spinal cord transection was selected to evaluate the effect of bFGF/HEMA-MOETACL implantation on SCI in vivo. Forty young adult Sprague–Dawley rats (220–250 g) were purchased from the Experimental Animal Center of Shaanxi Province (Xi'an, PR China). The experimental protocol was approved by the Xi'an Jiaotong University Laboratory Animal Administration Committee, performed according to the Xi'an Jiaotong University Guidelines for Animal Experimentation and conformed to the Guide for the Care and Use of Laboratory Animals published by the US National Institutes of Health. Rats were positioned on a cork platform after being anesthetized with 10% chloral hydrate (3.5 mL/kg, i.p.). Atropine (0.2 mL administered subcutaneously) and gentamicin (0.05 mL administered intramuscularly) were administered preoperatively. For each rat, the skin was incised along the midline of the back and the vertebral column was exposed. A T-9 laminectomy was performed under a surgical microscope using aseptic surgical techniques. The dura mater was opened and a segment of spinal cord, 2 mm in length, was dissected out to produce the SCI. Using a surgical microscope, we ensured that no remaining tissue was left in this segment.

In 8 rats the dura was sutured using 10-0 Ethilon (Ethicon, Johnson & Johnson), followed by muscle and skin closure. These rats without implantation treatment received aCSF only and served as the lesion-control group. In the remaining rats, we reopened the surgical site 5 days later and removed the debris from the lesion. In 12 rats, we inserted a bFGF/HEMA-MOETACL transplant, followed by suturing of the dura, muscles and skin. These animals served as the HEMA-MOETACL hydrogel + bFGF treatment group (HEMA-MOETACL + bFGF group). In 10 rats, we implanted a block of aCSF-incorporated HEMA-MOETACL hydrogel surrounded by acellular vascular matrix into the lesion. The dura was closed, followed by suturing of the muscle and skin. These animals served as the HEMA-MOETACL hydrogel-alone treatment group (HEMA-MOETACL-alone group). In 10 rats, we injected drug solution into the lesion, followed by suturing of the dura, muscles and skin. These animals served as the bFGF-alone treatment group (bFGF-alone group). In all 4 groups, bladder evacuation was performed twice a day until recovery of sphincter control and gentamicin was administered intramuscularly for 5 days to prevent urinary infection. All animals were kept in cages and received food and water ad libitum.

### Behavioral studies

Following implantation, locomotor skills were monitored using the Basso, Beattie and Bresnahan (BBB) open field locomotor testing procedure described previously^49^, once a week for 8 weeks. Rats were observed walking freely in an open field and the BBB locomotor rating scale was assessed by two blinded observers according to recovery of hindlimb movements. Sensorimotor skills were monitored using the grid navigation test^50^. This test was used to assess the deficit in descending motor control. Following implantation, rats had to cross a 1 m long grid with round metal bars separated by 4 cm 5 times, once a week for 8 weeks. A score was attributed by two blinded observers according to recovery of hindlimb movements.

### Electrophysiological studies

Eight weeks after implantation, the rats were re-anesthetized. As previously reported^51^, electrophysiological studies were performed prior to tissue harvesting. The spinal cord repair area was insulated from the surrounding muscle with a rubber dam. A bipolar stimulating electrode was placed in the motor cortex. A recording electrode was placed in the contralateral sciatic nerve. The motor evoked potentials (MEPs) were then recorded with a PowerLab 4SP distal data acquisition system (Keypoint 3.02 Denmark). The bipolar stimulating electrode was placed in the contralateral sciatic nerve. The recording electrode was placed in the sensorimotor cortex. The somatosensory evoked potentials (SSEPs) were then recorded. Each SSEP or MEP consisted of an average of 300 single sweep epochs.

### Tissue processing and histological analyses

The rats were killed by an overdose of anesthetic eight weeks after implantation. They were then perfused with physiological saline followed by 4% paraformaldehyde in 0.1 M PBS (pH 7.4). The spinal cords were carefully excised and postfixed in the same fixative at 4°C for 6–8 h and stored in 30% sucrose in 0.1 M PBS (pH 7.4) overnight. Next, the samples were examined under a dissecting microscope and 3-cm long segments of the thoracic region of the spinal cords including injury epicenters with implanted hydrogels were carefully dissected out, embedded in O.C.T. compound (Sakura Tokyo, Japan) and cut into 10-mm sections using a cryostat. The longitudinal sections were stained with HE using standard protocols and the slides were specifically evaluated, using a light microscope, for an adverse foreign-body type granulomatous reaction and the presence of connective and nervous tissue elements inside the hydrogel. For immunofluorescence studies, the following primary antibodies and dilutions were used: anti-glial fibrillary acidic protein (GFAP)-Cy3 (IgM, 1:200, Sigma-Aldrich) to identify astrocytes, anti-NF 160 (IgM, 1:200, Sigma-Aldrich) to identify neurofilaments (axons/dendrites) and anti-RECA-1 (IgM, 1:50, Abcam) to identify blood vessel endothelial cells. Cy3-conjugated anti-mouse IgM (1:100, Chemicon International) was used as a secondary antibody. GFAP-positive astrocytes, NF 160-positive neurofilaments and RECA-1-positive blood vessel endothelial cells inside/outside the hydrogel were visualized in longitudinal sections using a fluorescence microscope. Control sections in which the primary antibodies were omitted were routinely prepared to check for non-specific staining.

### Cavity measurement

HE stained sections were examined to measure the cavity volume. For morphometry, every fifth section was selected and its image was captured using a digital camera; high-resolution images were used to trace the areas of the cavities. The identified areas in individual sections were measured using image analysis software (Neurolucida, version 5.05.4). Any necrotic tissue within the cavities was counted as part of the lesion. The cavity volume was assessed as the sum of section areas multiplied by the distance between them and the total spinal cord volume of the segment was also measured^52^. The percentage cavitation (%Vol_cav_) was determined according to the following equation: %Vol_cav_ = Vol_cav_/Vol_total_ × 100%.

### Quantitative analyses and statistical analysis

Infiltration of the hydrogel due to ingrowth of neural processes and blood vessels was evaluated in the peripheral zone and the central zone of the hydrogel. Ingrowth was quantified using 6 slices from each spinal cord on average. Each calculation was divided by the area of the hydrogel implant (expressed in percentage) as the hydrogel did not always perfectly match the shape and size of the analysis window. Thus, the amount of ingrowth of axons and blood vessels was expressed as a percentage of the total area.

The mean values were reported as mean ± standard error of the mean (SEM). The data were analyzed using one-way analysis of variance (ANOVA) and Student's *t*-test with the SPSS16.0 software package (SPSS Inc., Chicago, IL, USA). Values of *p* < 0.05 were considered statistically significant.

## References

[CR1] Fehlings MG (2001). Editorial: recommendations regarding the use of methylprednisolone in acute spinal cord injury: making sense out of the controversy. Spine.

[CR2] Tator CH (2006). Review of treatment trials in human spinal cord injury: issues, difficulties and recommendations. Neurosurgery.

[CR3] Hawryluk GW, Rowland J, Kwon BK, Fehlings MG (2008). Protection and repair of the injured spinal cord: a review of completed, ongoing and planned clinical trials for acute spinal cord injury. Neurosurg Focus.

[CR4] Woerly S, Doan VD, Sosa N, Vellis JD, Espinosa A (2001). Reconstruction of the transected cat spinal cord following NeuroGel implantation: axonal tracing, immunohistochemical and ultrastructural studies. Int J Dev Neurosci.

[CR5] Teng YD (2002). Functional recovery following traumatic spinal cord injury mediated by a unique polymer scaffold seeded with neural stem cells. Proc Natl Acad Sci USA.

[CR6] Schmidt CE, Leach JB (2003). Neural tissue engineering: strategies for repair and regeneration. Annu Rev Biomed Eng.

[CR7] Hejčl A (2008). Biocompatible hydrogels in spinal cord injury repair. Physiol Res.

[CR8] Lesný P (2002). Polymer hydrogels usable for nervous tissue repair. J Chem Neuroanat.

[CR9] Lesný P (2006). Macroporous hydrogels based on 2-hydroxyethyl methacrylate. Part 4: Growth of rat bone marrow stromal cells in three-dimensional hydrogels with positive and negative surface charges and in polyelectrolyte complexes. J Mater Sci Mater Med.

[CR10] Prádný M, Petrovický P, Fronková V, Vacík J, Smetana K (2002). Evaluation of biocompatibility of the copolymer of 2-hydroxyethyl methacrylate with 2-(methylsulfanyl)ethyl methacrylate. J Mater Sci Mater Med.

[CR11] Lee TT, Green BA, Dietrich WD, Yezierski RP (1999). Neuroprotective effects of basic fibroblast growth factor following spinal cord contusion injury in the rat. J Neurotrauma.

[CR12] Ziche M, Morbidelli L (2000). Nitric oxide and angiogenesis. J Neurooncol.

[CR13] Beenken A, Mohammadi M (2009). The FGF family: biology, pathophysiology and therapy. Nat Rev Drug Discov.

[CR14] Mayer E, Dunnett SB, Pellitteri R, Fawcett JW (1993). Basic fibroblast growth factor promotes the survival of embryonic ventral mesencephalic dopaminergic neurons-I. Effects in vitro. Neuroscience.

[CR15] Kang CE, Baumann MD, Tator CH, Shoichet MS (2013). Localized and sustained delivery of fibroblast growth factor-2 from a nanoparticle-hydrogel composite for treatment of spinal cord injury. Cells Tissues Organs.

[CR16] Karimi-Abdolrezaee S, Eftekharpour E, Wang J, Schut D, Fehlings MG (2010). Synergistic effects of transplanted adult neural stem/progenitor cells, chondroitinase and growth factors promote functional repair and plasticity of the chronically injured spinal cord. J Neurosci.

[CR17] Liu WG, Wang ZY, Huang ZS (2011). Bone marrow-derived mesenchymal stem cells expressing the bFGF transgene promote axon regeneration and functional recovery after spinal cord injury in rats. Neurol Res.

[CR18] Greenwald SE, Berry CL (2000). Improving vascular grafts: the importance of mechanical and haemodynamic properties. J Pathol.

[CR19] Schmidt CE, Baier JM (2000). Acellular vascular tissues: natural biomaterials for tissue repair and tissue engineering. Biomaterials.

[CR20] Conklin BS, Richter ER, Kreutziger KL, Zhong DS, Chen C (2002). Development and evaluation of a novel decellularized vascular xenograft. Med Eng Phys.

[CR21] Hilbert SL, Boerboom LE, Livesey SA, Ferrans VJ (2004). Explant pathology study of decellularized carotid artery vascular grafts. J Biomed Mater Res A.

[CR22] Hejčl A (2009). Macroporous hydrogels based on 2-hydroxyethyl methacrylate. Part 6: 3D hydrogels with positive and negativesurface charges and polyelectrolyte complexes in spinal cord injury repair. J Mater Sci Mater Med.

[CR23] Hejčl A (2008). Acute and delayed implantation of positively charged 2-hydroxyethyl methacrylate scaffolds in spinal cord injury in the rat. J Neurosurg Spine.

[CR24] Bakshi A (2004). Mechanically engineered hydrogel scaffolds for axonal growth and angiogenesis after transplantation in spinal cord injury. J Neurosurg Spine.

[CR25] Tsai EC, Dalton PD, Shoichet MS, Tator CH (2004). Synthetic hydrogel guidance channels facilitate regeneration of adult rat brainstem motor axons after complete spinal cord transection. J Neurotrauma.

[CR26] Lampe KJ, Mooney RG, Bjugstad KB, Mahoney MJ (2010). Effect of macromer weight percent on neural cell growth in 2D and 3D nondegradable PEG hydrogel culture. J Biomed Mater Res A.

[CR27] Seidlits SK (2010). The effects of hyaluronic acid hydrogels with tunable mechanical properties on neural progenitor cell differentiation. Biomaterials.

[CR28] Bellamkonda R, Ranieri JP, Bouche N, Aebischer P (1995). Hydrogel-based three-dimensional matrix for neural cells. J Biomed Mater Res.

[CR29] Hejčl A (2013). Adjusting the chemical and physical properties of hydrogels leads to improved stem cell survival and tissue ingrowth in spinal cord injury reconstruction: a comparative study of four methacrylate hydrogels. Stem Cells Dev.

[CR30] Urdzíková L (2006). Transplantation of bone marrow stem cells as well as mobilization by granulocyte-colony stimulating factor promotes recovery after spinal cord injury in rats. J Neurotrauma.

[CR31] Syková E (2006). Autologous bone marrow transplantation in patients with subacute and chronic spinal cord injury. Cell Transplant.

[CR32] Zhang HY (2013). Regulation of autophagy and ubiquitinated protein accumulation by bFGF promotes functional recovery and neural protection in a rat model of spinal cord injury. Mol Neurobiol.

[CR33] Gupta D, Tator CH, Shoichet MS (2006). Fast-gelling injectable blend of hyaluronan and methylcellulose for intrathecal, localized delivery to the injured spinal cord. Biomaterials.

[CR34] Rabchevsky AG (2000). Basic fibroblast growth factor (bFGF) enhances functional recovery following severe spinal cord injury to the rat. Exp Neurol.

[CR35] Relf M (1997). Expression of the angiogenic factors vascular endothelial cell growth factor, acidic and basic fibroblast growth factor, tumor growth factor beta-1, platelet-derived endothelial cell growth factor, placenta growth factor and pleiotrophin in human primary breast cancer and its relation to angiogenesis. Cancer Res.

[CR36] Cuevas P (1991). Hypotensive activity of fibroblast growth factor. Science.

[CR37] Jones LL, Tuszynski MH (2001). Chronic intrathecal infusions after spinal cord injury cause scarring and compression. Microsc Res Tech.

[CR38] Parr AM, Tator CH (2007). Intrathecal epidermal growth factor and fibroblast growth factor-2 exacerbate meningeal proliferative lesions associated with intrathecal catheters. Neurosurgery.

[CR39] Coumans JV (2001). Axonal regeneration and functional recovery after complete spinal cord transection in rats by delayed treatment with transplants and neurotrophins. J Neurosci.

[CR40] Woerly S, Doan VD, Evans-Martin F, Paramore CG, Peduzzi JD (2001). Spinal cord reconstruction using NeuroGel implants and functional recovery after chronic injury. J Neurosci Res.

[CR41] Zai LJ, Yoo S, Wrathall JR (2005). Increased growth factor expression and cell proliferation after contusive spinal cord injury. Brain Res.

[CR42] Lee YL, Shih K, Bao P, Ghirnikar RS, Eng LF (2000). Cytokine chemokine expression in contused rat spinal cord. Neurochem Int.

[CR43] Loy DN (2002). Temporal progression of angiogenesis and basal lamina deposition after contusive spinal cord injury in the adult rat. J Comp Neurol.

[CR44] Glaser J, Gonzalez R, Sadr E, Keirstead HS (2006). Neutralization of the chemokine CXCL10 reduces apoptosis and increases axon sprouting after spinal cord injury. J Neurosci Res.

[CR45] Prádný M (2005). Macroporous hydrogels based on 2-hydroxyethyl methacrylate. Part II. Copolymers with positive and negative charges, polyelectrolyte complexes. J Mater Sci Mater Med.

[CR46] Cho SW (2005). Vascular patches tissue-engineered with autologous bone marrow-derived cells and decellularized tissue matrices. Biomaterials.

[CR47] Yang CF (2000). Experimental corneal neovascularization by basic fibroblast growth factor incorporated into gelatin hydrogel. Ophthalmic Res.

[CR48] Furuya T (2013). Treatment with basic fibroblast growth factor-incorporated gelatin hydrogel does not exacerbate mechanical allodynia after spinal cord contusion injury in rats. J Spinal Cord Med.

[CR49] Basso DM, Beattie MS, Bresnahan JC (1995). A sensitive and reliable locomotor rating scale for open field testing in rats. J Neurotrauma.

[CR50] Quertainmont R (2012). Mesenchymal stem cell graft improves recovery after spinal cord injury in adult rats through neurotrophic and pro-angiogenic actions. PLoS One.

[CR51] Choi JS (2012). Effects of human mesenchymal stem cell transplantation combined with polymer on functional recovery following spinal cord hemisection in rats. Korean J Physiol Pharmacol.

[CR52] Hains BC, Saab CY, Lo AC, Waxman SG (2004). Sodium channel blockade with phenytoin protects spinal cord axons, enhances axonal conduction and improves functional motor recovery after contusion SCI. Exp Neurol.

